# Oxidant Antioxidants and Adaptive Responses to Exercise

**DOI:** 10.1155/2015/290190

**Published:** 2015-03-24

**Authors:** Paola Venditti, Mari Carmen Gomez-Cabrera, Yong Zhang, Zsolt Radak

**Affiliations:** ^1^Dipartimento di Biologia, Università di Napoli “Federico II”, Complesso Universitario Monte Sant'Angelo, Edificio 7, Via Cinthia 21, 80126 Napoli, Italy; ^2^Department of Physiology, Faculty of Medicine, University of Valencia, Avenida Blasco Ibañez 15, 46010 Valencia, Spain; ^3^Department of Health and Exercise Science, Tianjin University of Sport, 51 Weijin South Road, Hexi District, Tianjin 300381, China; ^4^University of Physical Education, Alkotas Utca 44, Budapest 1123, Hungary

The extensive damage produced by unaccustomed (acute) exercise and the health benefits of regular physical activity are well-known phenomena as well as the role played in them by reactive oxygen species (ROS).

The present issue reports some interesting studies showing that the Janus faced effects of exercise-induced ROS in skeletal muscle.

Most studies dealing with ROS contribution to acute exercise-induced tissue damage determine the levels of markers of oxidative damage to specific substances but they do not take into account total redox status of an individual before and after exercise. In their research article D. Stagos et al. used markers measuring plasma static (sORP) and capacity (cORP) of oxidation-reduction potential (ORP) and more common redox markers for evaluating the effect of eccentric exercise. They showed that only in individuals exhibiting decrease in cORP after exercise, there were increases in lipid peroxidation, suggesting that cORP decrease after exercise can be indicative of oxidative stress induction.

Acute exercise-induced damage can be prevented by supplementation with single antioxidants. On the other hand, several studies suggest that a diet containing a mixture of different antioxidants is more effective in prevention of diseases dependent on high ROS production. In their paper, S. Carfagna et al. used a diet supplemented with* G. sulphuraria,* an alga exhibiting high antioxidant properties, mainly due to high content GSH and phycobilin proteins, known for their capacity to protect membranes from oxidative attack, for animals subjected to acute exercise. It was observed that* G. sulphuraria* prevented the oxidative stress in various tissues and preserved mitochondrial functionality, thus showing that dietetic integration with a source rich in antioxidants offers high protection against exercise-induced oxidative stress.

Mitochondrial respiratory chain is considered a major source of ROS during exercise, even though underlying mechanisms remain to be completely elucidated. In their work P. Wang et al. showed that, during exercise, there was a time-dependent increase in mitochondrial ROS production, which was correlated with upregulation of p66^shc^ (a protein involved in H_2_O_2_ generation) and FOXO3a (a factor activating the transcription of mitochondrial superoxide dismutase and catalase). The authors suggest that the ROS production stimulated by p66^shc^ is counteracted by catalase transcription by FOXO3a.

In human athletes, S. Mrakic-Sposta et al. evaluated the effects of a high-intensity discontinuous training period on ROS production and reducing capacity (assessed in capillary blood by EPR and redox sensor, resp.) after acute exercise. They showed that acute exercise induced a ROS increase dependent on exercise intensity, which was reduced by training confirming that this offers protection against oxidative stress induced by acute exercise.

Regular physical activity is associated with many health benefits including reduced risk for disorders such as cardiovascular disease, cancer, and diabetes and in the promotion of healthy aging. The training-induced cardiovascular protection has been investigated by A. Pósa et al., which showed that the training preservation against cardiac injury might be associated with the decreased activity of matrix metalloproteinases 2.

In another paper, A. Pósa et al. showed that physical exercise, associated with caloric restriction, is able to prevent the metabolic syndrome induced by high calorie diet.

Despite diversity, it is our believe that the articles comprised in this special issue could represent an important contribution to improve the knowledge of the mechanisms that are verified during physical activity and training and that make the exercise an ideal strategy in the prevention and treatment of many pathological conditions.

*Paola Venditti* *Paola Venditti* *Mari Carmen Gomez-Cabrera* *Mari Carmen Gomez-Cabrera* *Yong Zhang* *Yong Zhang* *Zsolt Radak* *Zsolt Radak*

